# Human papillomavirus infection in Bhutan at the moment of implementation of a national HPV vaccination programme

**DOI:** 10.1186/1471-2334-14-408

**Published:** 2014-07-22

**Authors:** Ugyen Tshomo, Silvia Franceschi, Dorji Dorji, Iacopo Baussano, Vanessa Tenet, Peter JF Snijders, Chris JLM Meijer, Maaike CG Bleeker, Tarik Gheit, Massimo Tommasino, Gary M Clifford

**Affiliations:** 1 Department of Obstetrics & Gynaecology, Jigme Dorji Wangchuck National Referral Hospital, Thimphu, Bhutan; 2 International Agency for Research on Cancer, 150 cours Albert Thomas, 69372 Lyon, cedex 08, France; 3 Department of Laboratory Services, Jigme Dorji Wangchuck National Referral Hospital, Thimphu, Bhutan; 4 VU University Medical Center, De Boelelaan 1117, 1081 HV Amsterdam, the Netherlands

**Keywords:** Human papillomavirus, Prevalence, Cervical cancer, Bhutan

## Abstract

**Background:**

Cervical cancer is the most common female cancer in Bhutan, the first low/middle-income country to implement a national human papillomavirus (HPV) vaccination programme.

**Methods:**

To provide a robust baseline for future evaluations of vaccine effectiveness, cervical cell specimens were obtained from 2,505 women aged 18–69 years from the general population, and biopsies from 211 cervical intraepithelial neoplasia grade 3 (CIN3) and 112 invasive cervical cancer (ICC) cases. Samples were tested for HPV using GP5+/6+ PCR.

**Results:**

Among the general population, HPV prevalence was 26%, being highest (33%) in women ≤24 years, but remaining above 15% in all age-groups. Determinants of HPV included age, marital status, and number of sexual partners. Among the eight percent with cytological abnormalities, 24 CIN3 and 4 ICC were histologically confirmed. Even after additional testing with a sensitive E7 PCR, no infections with vaccine-targeted HPV types were detected in the few vaccinated women (n = 34) compared to 6% prevalence in unvaccinated women of similar age (p = 0 · 215).

**Conclusion:**

Based upon type-specific prevalence among biopsies, at least 70% of ICC in Bhutan are theoretically preventable by HPV16/18 vaccination, but screening programmes should be expanded among older women, who have an important underlying burden of CIN3 and ICC.

## Background

In the Himalayan kingdom of Bhutan, cervical cancer represents the most common cancer among females, with an age-standardised incidence rate of approximately 13 cases per 100,000 person years [1], one of the highest in Asia. Given such a high burden of disease and the limited coverage of cervical screening, Bhutan became the first low- or middle-income country (LMIC) to initiate a national vaccination programme against human papillomavirus (HPV), the necessary cause of cervical cancer. In 2010, over 130,000 doses of quadrivalent (HPV16/18/6/11) HPV vaccine were administered, primarily at schools. The 3 dose vaccination coverage is estimated at 92% among 12–18 year old girls [2]. Since 2011, 12-year old girls have continued to be vaccinated.

The impact of the vaccine programme on cervical cancer will not be seen for at least 20 years and, therefore, short-term evidence of vaccine programme effectiveness is crucial to encourage national planners to sustain HPV vaccination services. Hence, the Bhutan Ministry of Health, in collaboration with the International Agency for Research on Cancer (IARC), initiated a series of studies to evaluate the short term impact of HPV vaccine on HPV prevalence.

As no data existed on the burden of HPV among women in Bhutan, a baseline study of the prevalence of, and risk factors for, HPV infection prior to vaccination was initiated in 2011 according to the standardised protocol of the IARC HPV prevalence surveys [3]. Cervicovaginal samples were collected from women aged 18–69 years living in the capital, Thimphu, with a particular effort to oversample young (≤25 years) women. In addition, biopsy/tissue specimens were collected from women diagnosed with cervical intraepithelial neoplasia grade 3 (CIN3) and invasive cervical cancer (ICC), for HPV detection and genotyping.

## Methods

### General female population

Between December 2011 and October 2012, a survey was conducted by the Ministry of Health of Bhutan in collaboration with the IARC, Lyon, France. The study aim was to enrol 2,500 women from the general population using an age-stratified approach, namely 1,000 women aged below 25 years old, 500 women aged 25 to 29 years old, 200 women in each five-year age group between 30–34 and 45–49 years, and 200 women aged ≥50 years. Over-representation of young women was done in order to allow robust assessment of vaccine-induced changes in HPV prevalence in a repeat survey after 5 years. All mentally and physically competent women were eligible for the study, regardless of their marital status.

Study procedures were performed in the Reproductive Health Departments of Jigme Dorji Wangchuck National Referral Hospital (JDWNRH) in Thimphu, and in the hospital of Lungtenphu, a satellite town of Thimphu. To reach a broad and representative sample of the population living around the two hospitals, women were recruited in two ways: 1) Women residing in the pre-defined areas surrounding the two hospitals were visited at home by social workers and invited to join the study. Participation among the 1217 and 673 women invited from Thimphu and Lungtenphu was 25.7% and 54.2%, respectively. 2) In addition, women consulting outpatient clinics in the two hospitals (mainly antenatal care or family planning clinics in which cervical cancer screening is also offered to women aged 20 or older), were also invited to join. Participation among women invited through the hospital was close to 100%.

Some limited sociodemographic information was obtained from all women invited from home in Thimphu, among whom non-participants tended to be younger and more likely to be unmarried in comparison to participants. Following signature of an informed consent form, a structured questionnaire including information on socio-demographic characteristics, sexual behaviour of the women and of their partners, reproductive factors, use of contraceptive methods and smoking habits was administered to all study participants. History of HPV vaccination was asked to women aged ≤20 years.

A cytobrush (Cervex-Brush, Rovers Medical Devices B.V., The Netherlands) was used for the collection of exfoliated cervical cells from the endocervix and ectocervix. After preparation of a conventional Pap smear, the brush containing cellular material was placed in a vial containing PreservCyt medium (Cytyc, Boxbourough, MA, USA) and stored at +4C until shipment.

Conventional Pap smears were read in the Department of Cytology, JDWNRH, and reported according to the 2001 Bethesda System [4]. All women with abnormal cervical findings were recalled to have a colposcope-aided examination and, if necessary, a colposcopically-directed biopsy and appropriate treatment by local gynaecologists. Histological confirmation of cervical tissue was performed at JDWNRH.

### Women with CIN3 and ICC

Formalin-fixed paraffin-embedded biopsies were retrieved from all cases of ICC and CIN3 diagnosed between 2010 and 2012 at JDWNRH, Thimphu or the Eastern Regional Referral Hospital, Mongar. Biopsies were sent to the VU University Medical Center, Amsterdam, for HPV DNA testing and review of histology. After exclusion of 11 biopsies negative for beta-globin PCR and 177 biopsies without any histological evidence of CIN3 or cancer, 211 CIN3 and 112 ICC cases were included in following analyses (of which 17 CIN3 and 4 ICC, respectively, derived from women included in the general population survey described above).

### HPV testing and genotyping

HPV testing was performed on all exfoliated cervical cells and biopsies in the Department of Pathology at the VU University Medical Center, Amsterdam. DNA was extracted from the PreservCyt sample using magnetic beads (Macherey-Nagel) on a robotic system (Hamilton Robotics) according to the manufacturer’s instructions. Biopsies were sectioned using a ‘sandwich’ approach, whereby inner tumour sections were destined for HPV testing and outer sections for histological confirmation of tumour tissue (see above). One or more sections representing approximately 1 cm^2^ of tissue were predigested with proteinase K after which DNA was extracted using magnetic beads (Macherey-Nagel).

Beta-Globin PCR analysis was conducted first to confirm the presence of human DNA in all specimens [5]. The presence of HPV DNA was determined by conducting a general primer GP5+/6+ - mediated PCR, which permits the detection of a broad spectrum of genital HPV types [6]. HPV positivity was assessed by hybridization of PCR products in an enzyme immunoassay with 2 oligoprobe cocktails that, together, detect the following 44 mucosal HPV types: HPV6, 11, 16, 18, 26, 30, 31, 32, 33, 34, 35, 39, 40, 42, 43, 44, 45, 51, 52, 53, 54, 55, 56, 57, 58, 59, 61, 64, 66, 67, 68, 69, 70, 71, 72, 73, 81, 82, 83, 84, 85, 86, 89, and 90. Subsequent HPV genotyping was conducted by reverse-line blot hybridization of GP5+/6+ PCR products [7]. HPV types considered high-risk (HR) types for this analysis comprised HPV16, 18, 31, 33, 35, 39, 45, 51, 52, 56, 58, 59, and 68 [8]; possible HR types comprised HPV26, 30, 34, 53, 66, 67, 69, 70, 73, 82, and 85; all other HPV types were considered low-risk (LR).

DNA extracted from cervical cells of women aged ≤ 24 years, was additionally tested for the 13 HR types plus HPV6 and 11 using an alternative PCR-based assay based upon multiplex E7 gene PCR and DNA microarray [9] at IARC. This assay has been validated against a WHO HPV LabNet proficiency panel [10], and is known to be more sensitive than GP5+/6 + -mediated PCR in detecting low copy numbers of viruses, particularly in the presence of multiple infections [11].

### Statistical analyses

In addition to crude prevalence, age-standardised HPV prevalence was computed using the world standardised population to allow comparisons of HPV prevalence with other IARC surveys [3]. Prevalence ratios (PR) for HPV positivity and corresponding 95% confidence intervals (CI) were computed using two binomial regression models with a log link, the first adjusted for the study design variables age group (<24; 25–29, 30–34, 35–39, 40–44, and >45 years) and hospital where examined (JDWNRH or Lungthenphu), and a second model that adjusted additionally for lifetime sexual partners. Risk trends were assessed by considering categories as continuous variables. PRs were also used to compare HPV type-specific prevalence in HPV-positive ICC and normal cytology.

### Ethical approval

The present study had the approval of both the Research Ethical Board of the Bhutan Ministry of Health and the IARC Ethics Committee.

## Results

### General female population

Of the 2,525 participants who provided cervical cell samples, 2,505 had valid HPV results and were included in the following analyses, including 1,638 and 867 recruited at JDWNRH and Lungthenphu hospitals, respectively. Among the 2,468 women with a valid PAP smear result, 196 (8%) had an abnormal cytological diagnosis, including 123 (58%) with atypical squamous cells of undetermined significance, atypical squamous cells cannot exclude high-grade lesion or atypical glandular cells of undetermined significance (ASCUS/ASC-H/AGUS), 59 (2%) low-grade squamous intraepithelial lesions (LSIL), and 14 (1%) high-grade squamous intraepithelial lesions or worse (HSIL+). Histological confirmation was obtained for 135 women with abnormal cytology, among whom 23 CIN2, 24 CIN3 and 4 ICC were histologically confirmed.

**Figure 1:**
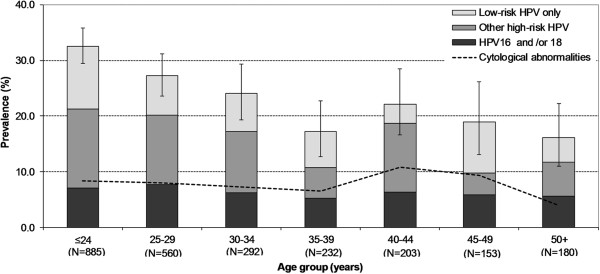
**Age-specific prevalence of human papillomavirus (HPV) DNA and of cytological abnormalities among 2,505 women.** Bhutan, 2011–2012. HPV = human papillomavirus.

Overall HPV prevalence was 26% (95% CIs: 24–28), being 23% (95% CIs: 21–25) among women with normal cytology (Table 1). Corresponding overall HPV prevalence age-standardised to the world population was 27%. The prevalence of HPV types in abnormal cytology was 61% (Table 1), being 52%, 81%, and 57% in ASCUS/ASC-H/AGUS, LSIL and HSIL+, respectively. In total, 437 (17%) women had single-type and 218 (9%) had multiple-type infections. Crude and age-standardised prevalence of HR types was 18%. Crude prevalence of possible HR HPV was 5%. HPV16 was the most common HPV type both in women with normal (3%) and abnormal (14%) cytology.Figure 1 shows the age-specific prevalence of HPV and cytological abnormalities, classified hierarchically into (1) HPV16 or 18, (2) other HR types and (3) LR types only. Overall HPV prevalence was highest (33%, 95% CI: 30–36) among women <24 years, and decreased with age, down to 16% (95% CI: 11–22) among women aged ≥50 years. Prevalence of cytological abnormalities varied between 4% in women aged ≥50 years and 11% in women aged 40–44 years. The prevalence of CIN3 or worse was about 1% in all age-groups, but all 4 ICC were diagnosed only in women aged over 35 years old (data not shown).

**Table 1:** Prevalence of human papillomavirus (HPV) types by cytological findings and overall among 2,505 women in Bhutan, 2011-2012

**HPV type**	**Normal (n = 2272)**				**Abnormal (n = 196)**				**Total (n = 2505)**^**a**^			
	**Single**	**Multiple**	**Total**	**(%)**	**Single**	**Multiple**	**Total**	**(%)**	**Single**	**Multiple**	**Total**	**(%)**
**HPV-**	-	-	1750	(77)	-	-	76	(39)	-	-	1850	(74)
**HPV+**	358	164	522	(23)	70	50	120	(61)	437	218	655	(26)
***High-risk***												
**16**	48	30	78	(3.4)	15	13	28	(14)	65	44	109	(4.4)
**18**	26	27	53	(2.3)	2	9	11	(5.6)	29	37	66	(2.6)
**31**	14	7	21	(0.9)	4	3	7	(3.6)	19	10	29	(1.2)
**33**	9	12	21	(0.9)	6	4	10	(5.1)	16	17	33	(1.3)
**35**	3	6	9	(0.4)	3	2	5	(2.6)	6	8	14	(0.6)
**39**	11	5	16	(0.7)	0	6	6	(3.1)	11	11	22	(0.9)
**45**	9	19	28	(1.2)	1	8	9	(4.6)	10	27	37	(1.5)
**51**	15	14	29	(1.3)	6	8	14	(7.1)	21	22	43	(1.7)
**52**	16	12	28	(1.2)	3	3	6	(3.1)	19	15	34	(1.4)
**56**	17	14	31	(1.4)	4	8	12	(6.1)	21	23	44	(1.8)
**58**	14	13	27	(1.2)	11	6	17	(8.7)	25	19	44	(1.8)
**59**	21	19	40	(1.8)	4	2	6	(3.1)	26	21	47	(1.9)
**68**	4	5	9	(0.4)	3	1	4	(2.0)	7	6	13	(0.5)
**Any**	214^b^	125	339^b^	(15)	62	42	104	(53)	282^b^	169	451^b^	(18)
***Possibly high-risk***												
**26**	1	1	2	(0.1)	0	0	0	(0.0)	1	1	2	(0.1)
**30**	3	7	10	(0.4)	1	1	2	(1.0)	4	8	12	(0.5)
**34**	0	1	1	(0.0)	0	0	0	(0.0)	0	1	1	(0.0)
**53**	2	4	6	(0.3)	1	2	3	(1.5)	3	6	9	(0.4)
**66**	4	13	17	(0.7)	0	3	3	(1.5)	4	16	20	(0.8)
**67**	15	24	39	(1.7)	1	9	10	(5.1)	17	33	50	(2.0)
**69**	0	3	3	(0.1)	0	1	1	(0.5)	0	4	4	(0.2)
**70**	8	13	21	(0.9)	0	2	2	(1.0)	8	15	23	(0.9)
**73**	1	5	6	(0.3)	0	2	2	(1.0)	1	7	8	(0.3)
**82**	2	2	4	(0.2)	1	1	2	(1.0)	3	3	6	(0.2)
**85**	0	1	1	(0.0)	0	1	1	(0.5)	0	2	2	(0.1)
**Any**	36	62	98	(4.3)	4	19	23	(11.7)	41	81	122	(4.9)
***Low-risk***												
**6**	8	5	13	(0.6)	1	0	1	(0.5)	9	5	14	(0.6)
**11**	6	4	10	(0.4)	1	1	2	(1.0)	7	5	12	(0.5)
**Other**	94	99	193	(9.4)	2	24	26	(13)	98	125	223	(8.9)
**Any**	108	106	214	(9.4)	4	25	29	(17)	114	133	247	(10)

^a^37 inadequate cytology are also included in the total.

^b^The total is not equal to the sum of the 13 high-risk because of 7 uncharacterized high-risk infections that are also included in the total.

Table 2 shows the relationship between HPV positivity and various characteristics of participants. In a model adjusted for age and hospital only, significant determinants of HPV positivity included age group (PR for ≥45 vs ≤24 years = 0 · 53; 95% CI: 0 · 42–0 · 69), hospital (PR for Lungthenphu versus JDWNRH = 0 · 81; 95% CI: 0 · 70-0 · 94), ethnic group (PR for Lhotsampa vs Scharchop = 0 · 69; 95% CI: 0 · 57–0 · 83), marital status (PR for separated/widowed versus married = 1 · 34; 95% CI: 1 · 04–1 · 74), number of marriages (PR for ≥2 vs 1 = 1 · 62; 95% CI: 1 · 32–1.99), number of lifetime sexual partners (PR for ≥3 vs 1 = 1 · 93; 95% CI: 1 · 21–3.08), number of pregnancies (PR for nulliparous vs 1 = 1 · 46; 95% CI: 1 · 23–1 · 74), age at first pregnancy (PR for ≤22 vs <19 = 0 · 78; 95% CI: 0 · 63–0 · 95), husbands extramarital relations (PR for Yes, during marriage = 1.71, 95% CI 1.27-2.31), use of any contraceptive method (PR for ever vs never = 0 · 80; 95% CI: 0 · 69–0 · 93), history of PAP smear (PR = 0 · 77; 95% CI: 0 · 66-0 · 90), receiving cash for sex (PR = 2 · 77; 95% CI: 1 · 55–4 · 95) or having genital warts (PR = 2 · 17; 95% CI: 1 · 06–4 · 45). All these associations remained significant in a second model additionally adjusted for lifetime number of sexual partners, with the exception of number of marriages.

**Table 2:** Prevalence ratios (PR) for human papillomavirus (HPV) positivity and corresponding 95% confidence intervals (CIs) according to selected characteristics among 2,505 women in Bhutan, 2011-2012

**Characteristic**	**N women**^**a**^	**HPV-positive (%)**	**Adjusted**^**b **^**PR**	**95% CI**	**Adjusted**^**c **^**PR**	**95% CI**
**Age-group (years)**						
≤24	885	289 (32.7)	1	-	1	-
25-34	852	223 (26.2)	0.82	0.71-0.95	0.81	0.70-0.94
35-44	435	85 (19.5)	0.61	0.49-0.75	0.57	0.46-0.71
≥45	333	58 (17.4)	0.53	0.42-0.69	0.51	0.39-0.65
*χ*^*2*^_*1*_*for trend*			*p < 0.001*		*p < 0.001*	
**Hospital where examined**						
Thimphu	1638	460 (28.1)	1	-	1	-
Lungthenphu	867	195 (22.5)	0.81	0.70-0.94	0.82	0.71-0.95
**Type of invitation**						
Clinic	1835	483 (26.3)	1	-	1	-
Door to door	670	172 (25.7)	1.06	0.91-1.24	1.08	0.92-1.26
**Ethnic group**						
Scharchop	949	264 (27.8)	1	-	1	-
Ngalop	586	180 (30.7)	1.11	0.95-1.30	1.11	0.94-1.30
Lhotsampa	585	116 (19.8)	0.69	0.57-0.83	0.70	0.57-0.85
Other	371	88 (23.7)	0.90	0.74-1.11	0.89	0.72-1.09
**Education (yrs)**						
Illiterate	1209	292 (24.2)	1	-	1	-
1-9	512	132 (25.8)	1.02	0.85-1.21	1.02	0.85-1.21
≥10	750	221 (29.5)	1.07	0.92-1.24	1.09	0.94-1.28
*χ*^*2*^_*1*_*for trend*			*p = 0.415*		*p = 0.274*	
**Marital status**						
Married	2298	586 (25.5)	1	-	1	-
Single	54	19 (35.2)	1.22	0.84-1.76	1.60	1.14-2.24
Separated/widowed	137	42 (30.7)	1.34	1.04-1.74	1.31	1.01-1.69
**Number of marriages**^**d**^						
1	2209	549 (24.9)	1	-	1	-
≥2	201	67 (33.3)	1.62	1.32-1.99	1.27	0.86-1.86
**Number of pregnancies**						
Nulliparous	276	117 (42.4)	1.46	1.23-1.74	1.50	1.26-1.78
1	776	228 (29.4)	1	-	1	-
2	588	134 (22.8)	0.85	0.70-1.03	0.83	0.68-1.01
≥3	839	163 (19.4)	0.84	0.67-1.05	0.81	0.65-1.01
*χ*^*2*^_*1*_*for trend*^e^			*p = 0.159*		*p = 0.090*	
**Age at first pregnancy**^**e**^						
<19	554	145 (26.2)	1	-	1	-
19-21	885	222 (25.1)	0.93	0.78-1.11	0.98	0.82-1.17
≥22	754	155 (20.6)	0.78	0.63-0.95	0.81	0.66-0.99
*χ*^*2*^_*1*_*for trend*			*p = 0.014*		*p = 0.041*	
**Contraceptive use**						
Never	562	173 (30.8)	1	-	1	-
Ever	1860	458 (24.6)	0.80	0.69-0.93	0.76	0.66-0.89
Hormonal only	853	233 (27.3)	0.82	0.69-0.96	0.78	0.66-0.92
Hormonal + other	276	63 (22.8)	0.79	0.61-1.02	0.74	0.57-0.95
Other only	777	174 (22.4)	0.78	0.65-0.94	0.75	0.63-0.90
**Lifetime sexual partners**^**f**^						
1	2228	566 (25.4)	1	-	1	-
2	180	60 (33.3)	1.54	1.24-1.92	1.54	1.24-1.92
≥3	26	10 (38.5)	1.93	1.21-3.08	1.93	1.21-3.08
*χ*^*2*^_*1*_*for trend*			*p < 0.001*		*p < 0.001*	
**Husbands “extramarital” sexual relationships**^**d**^						
No	1966	481 (24.5)	1	-	1	-
Yes, before marriage only	308	95 (30.8)	1.30	1.08-1.55	1.23	1.02-1.48
Yes, during marriage	78	28 (35.9)	1.71	1.27-2.31	1.49	1.09-2.05
Uncertain	76	22 (29.0)	1.02	0.72-1.47	1.00	0.70-1.43
**History of receiving cash for sex**^**g**^						
No	2467	641 (26.0)	1	-	1	-
Yes	9	5 (55.6)	2.77	1.55-4.95	-	-
**Last PAP smear (yrs)**						
Never	1367	418 (30.6)	1	-	1	-
Ever	1114	229 (20.6)	0.77	0.66-0.90	0.77	0.66-0.90
**Evidence of genital warts at visit**^**h**^						
No	2473	640 (25.9)	1	-	1	-
Yes	5	3 (60.0)	2.17	1.06-4.45	2.23	1.09-4.59

^a^Some figures do not add up to the total due to missing values.

^b^Adjusted for age and hospital as appropriate.

^c^Adjusted for age, hospital and lifetime sexual partners as appropriate.

^d^Among ever married women only.

^e^Among parous women only.

^f^3 virgins excluded.

^g^Model does not converge for PR adjusted for lifetime sexual partners.

^h^PR not adjusted for age because model does not converge.

**Figure 2:**
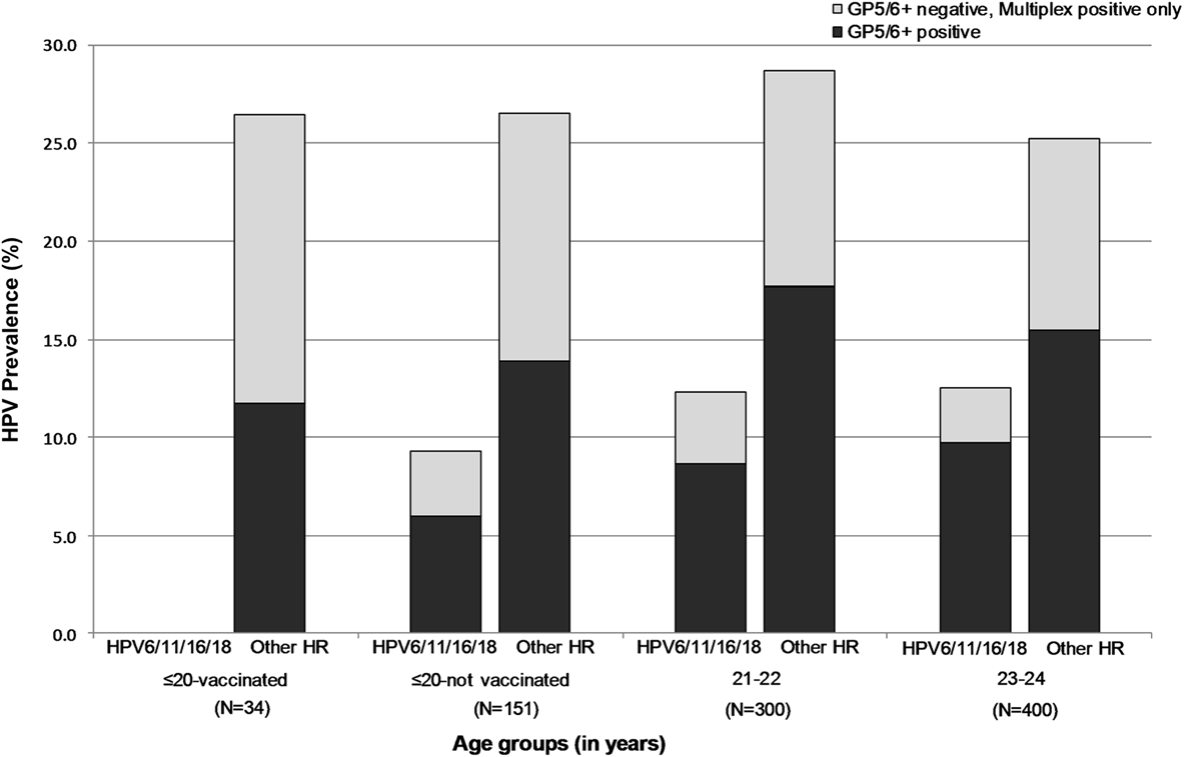
**Age-specific prevalence of human papillomavirus (HPV) DNA among 885 women ≤24 years old, according to two HPV DNA tests (GP5+/6+ and multiplex E7).** Bhutan, 2011–2012. HPV = human papillomavirus; HR = high-risk.

No associations with HPV positivity were seen for education level (49% of women reported no formal education), source of recruitment (27% from home, 73% from hospital) (Table 2), family income, smoking (11% ever smokers), chewing of non-tobacco products (36% ever chewers), occupation (67% housewife), age at menarche (median = 14 years), age at first sexual intercourse (median = 19 years), and difference in age with partner at first sexual intercourse (median = 4 years) (data not shown).Upon additional testing of women aged ≤24 years with multiplex E7 gene PCR (Figure 2), an additional 27 (3%) and 79 (9%) women became positive for vaccine-targeted HPV types and non-vaccine-targeted HR HPV types, respectively. Very few GP5+/6+ positive women tested negative by multiplex E7 PCR (2 and 13 for vaccine-targeted HPV types and non-vaccinated HR HPV types, respectively, data not shown). A small number of women aged ≤20 years reported HPV vaccination (n = 34), among whom the prevalence of non-vaccine targeted HR HPV types (12%) was similar to that seen in unvaccinated women of the same age, and that in women aged 21–24 (Figure 2). However, no infections with vaccine-targeted HPV types were detected in vaccinated women, compared to 6% in unvaccinated women of the same age (2-sided Fisher’s exact test, P = 0 · 215), and 9% in women aged 21–24 (p = 0 · 064).

### Women with CIN3 and ICC

Type-specific HPV prevalence is described in Table 3 for biopsies of 211 CIN3 and 112 ICC cases of which 87% and 88%, respectively, were HPV-positive. Type-specific HPV prevalence is reported among HPV-positive cases only and is compared to that among 523 HPV-positive women with normal cytology from the general population survey (Table 3). HPV16 was by far the most common HPV type, being detected in 60% and 63% of HPV-positive CIN3 and ICC, respectively. HPV16/18 and HPV16/18/31/33/45/52/58 together accounted for 70% and 88% of ICC, respectively. Upon comparison with HPV-positive women with normal cytology, PRs for women with ICC were 6.5 (95% CI: 4.5–9.4) for HPV16 and 6.7 (95% CI: 3.8-12.1) for HPV16/18/31/33/45/52/58, but did not exceed one for any other HR HPV types. Possible HR, LR and multiple-type infections were also much less common in ICC than in HPV-positive women with normal cytology (Table 3). HPV18 and HPV45 were detected in a larger proportion of ICC than CIN3, whereas the opposite was true for HPV58.

**Table 3:** Prevalence of selected human papillomavirus (HPV) types and groups of HPV types among HPV-positive women, by cervical status

**HPV type**	**Normal**^**a**^		**CIN3**^**b**^		**Cancer**^**b**^		**Cancer: normal cytology**
	**(N = 2272)**		**(N = 211)**		**(N = 112)**		**Prevalence ratio (95% CI)**
	**n**	**(% of HPV pos)**	**n**	**(% of HPV pos)**	**n**	**(% of HPV pos)**	
**Any**	522	-	183	-	99	-	-
**16**	78	(15)	109	(60)	62	(63)	5.8 (4.0-8.3)
**18**	53	(10)	4	(2)	7	(7)	0.7 (0.4-1.5)
**16/18**	127	(24)	113	(62)	69	(70)	5.0 (3.4-7.4)
**31**	21	(4)	14	(8)	4	(4)	1.0 (0.4-2.5)
**33**	21	(4)	12	(7)	2	(2)	0.5 (0.1-2.0)
**45**	28	(5)	2	(1)	5	(5)	0.9 (0.4-2.2)
**52**	28	(5)	2	(1)	3	(3)	0.6 (0.2-1.8)
**58**	27	(5)	32	(18)	5	(5)	1.0 (0.4-2.2)
**16/18/31/33/45/52/58**	235	(45)	168	(92)	87	(88)	6.7 (3.8-12.1)
**Other high-risk types**	104	(20)	11	(6)	9	(9)	0.4 (0.2-0.8)
**Possible high-risk types**	98	(19)	4	(2)	4	(4)	0.2 (0.1-0.6)
**Low-risk types**	214	(41)	8	(4)	5	(5)	0.1 (0.0-0.2)
**Multiple infections**	164	(31)	16	(9)	6	(6)	0.2 (0.1-0.4)

^a^HPV tested in cervical cells from women diagnosed with normal cytology from general population survey.

^b^HPV tested in biopsies from women diagnosed with histologically confirmed CIN3 and ICC from case series.

## Discussion

These data provide a picture of HPV infection in the adult female population of Bhutan just after the country embarked upon a highly successful national programme of HPV vaccination among 12–18 year old girls, to serve as a baseline for future impact monitoring. Data represent that of an almost entirely vaccine-free population and disclose a relatively high prevalence of HR HPV infection (18%), particularly among women younger than 25 years (22%). Findings can be confidently compared with other IARC HPV surveys that were conducted according to a similar sampling and HPV testing protocol. In this respect, the age-standardised HR HPV prevalence in Bhutan (18%) ranks below that of Guinea (31%) [12] and Mongolia (25%) [13], but is similar to the prevalence detected in Nigeria (18%) [14] and Vanuatu (19%) [15]. It is more elevated than that found in many other areas at high-risk for cervical cancer in Asia, including that in neighbouring countries of India (12%) [16], China (12-13%) [17-19] and Nepal (6%) [20]. HR HPV prevalence is strongly correlated internationally with cervical cancer incidence rates [21] and the high prevalence of cervical carcinoma (1 out of every 200 women aged 35+ years) in our study population is consistent with recent estimates of cervical cancer incidence in Bhutan [1].

HPV16 was by far the most frequently detected type in Bhutan, increasing from 3% of normal cytology up to 63% of HPV-positive ICC. Such a high proportion of HPV16 in cervical cancer agrees with findings of a meta-analysis in which the proportion of HPV16-positive ICC in Western/Central Asia (66%) was the highest among all world regions [19]. Infections with vaccine types HPV16 and/or 18 accounted for 24% of HR HPV positivity in the general female population and for 70% of cervical cancer, which gives an estimate of the potential future impact of the vaccine programme, if one simply assumes 100% efficacy and zero cross-protection against non-vaccine types. The proportion of ICC theoretically preventable by the seven HR HPV types (HPV16/18/31/33/45/52/58) included in a future nonavalent vaccine is 88%. Of note, HPV18 and HPV45 were under-represented in CIN3 in comparison to cancer, whereas certain HPV types, particularly HPV58, were responsible for a much larger proportion of CIN3 than of ICC. These mirror findings from other world regions in a recent meta-analysis [22].

Risk factors associated with cervical HPV infection were largely consistent with previous IARC HPV Surveys. Lifetime number of sexual partners was an important determinant of HPV positivity, although the mean reported number of women’s lifetime sexual partners (1.1) was relatively low. Being single, separated or divorced, as well as a history of receiving cash for sex (albeit rarely reported), were also positively associated with HPV prevalence. Nulliparous women had a significantly higher risk of being HPV-positive, even after adjustment, in agreement with a pooled analysis of previous IARC HPV Prevalence Surveys [23], but the linear trend by number of pregnancies was not significant among parous women. Use of both hormonal and non-hormonal contraceptives and condoms were associated with lower HPV prevalence in Bhutan, in contrast to previous findings of pooled analyses of the IARC HPV prevalence surveys that found no associations [23,24]. However, the effect of residual confounding by under-reported high-risk sexual behaviour on certain associations cannot be ruled out. Lastly, a lower prevalence (20%) was also remarked among the Lhotsampa ethnic group, who are an ethnically Nepalese and largely Hindu population. However, this prevalence remains somewhat higher than that observed in the IARC HPV prevalence survey in Nepal (9%) [20].

Forty-five percent of study participants reported to having a prior PAP smear, which gives an estimation of the coverage of the cytology-based programme that has been targeting women living in Thimphu since 1999. Cervical screening is unavailable, however, in most rural and higher-altitude areas of Bhutan. One possibility to improve nationwide coverage is the application of an HPV testing approach, e.g. careHPV in women aged 30 or older. Our data suggest that approximately 15% of screened women aged 30 or older might test positive for HR HPV and require diagnostic follow-up and treatment.

This study has weaknesses and strengths. In particular, the extent to which our study population is representative of the general female population in Thimphu, and Bhutan as a whole, is difficult to evaluate. However, HPV infection is asymptomatic and self-selection of women by level of HPV risk is unlikely to have occurred. Furthermore, there was no significant difference in HPV prevalence by whether women were recruited through hospital consultations or from their homes, neither was there any substantial difference in their distribution of any of the study variables in Table 1.

Strengths include the relatively large number of women, especially those younger than 25 years, and the high rate of colposcopical follow-up and histological confirmation for women with abnormal cytology, allowing a strong gold standard of disease ascertainment. Furthermore, we used two high-quality PCR tests of which one, GP5+/6+, is clinically validated and conducted in a central laboratory that allows comparability with other studies, notably previous IARC HPV Surveys.

We made a particular effort to sample a large number of women aged ≤24 years, and to test with a second highly-sensitive E7 multiplex PCR assay [9]. As expected, adding this test approximately doubled the HR HPV prevalence over that detected by GP5+/6+ [11]. The greater relative increase for non-vaccine-targeted than vaccine-targeted types is likely because the former have more mismatches with both GP-PCR primers [5].

This baseline survey represents the first part of a long-term protocol to monitor HPV vaccine impact in Bhutan, which will include a repeat HPV prevalence survey in cervicovaginal samples in 2016/17 among women aged ≤24 years to demonstrate the 5-year impact of the HPV vaccination programme, as well as repeated HPV prevalence surveys in urine samples of adolescents aged 17–19 years at high-schools in Thimphu, Bhutan.

## Conclusions

These data will serve as a robust baseline for future evaluations of HPV vaccine programme effectiveness on HPV prevalence as cohorts of vaccinated girls grow up. Nevertheless, our survey did also capture a few young vaccinated women in the baseline survey, among whom there was a very reassuring, albeit statistically weak, evidence of 100% protection against vaccine-targeted HPV type (HPV6/11/16/18) infection, irrespective of the HPV test. Whilst awaiting more statistically robust evaluations in the future, this ad hoc finding represents the first ever real-life evidence of HPV vaccination programme impact in a low- or middle-income country.

## Abbreviations

ASCUS/ASC-H/AGUS: Atypical squamous cells of undetermined significance, atypical squamous cells cannot exclude high-grade lesion or atypical glandular cells of undetermined significance; CI: Confidence interval; CIN: Cervical intraepithelial neoplasia; HR: High-risk; HPV: Human papillomavirus; HSIL: High-grade squamous intraepithelial lesions; IARC: International Agency for Research on Cancer; ICC: Invasive cervical cancer; JDWNRH: Jigme Dorji Wangchuck National Referral Hospital; LMIC: Low- and middle-income country; LR: Low-risk; LSIL: Low-grade squamous intraepithelial lesions; OR: Odds ratio; PR: Prevalence ratio.

## Competing interests

P.J.F. Snijders has Honoraria from Speakers Bureau from Roche and is a consultant/advisory board member for Roche and Gen-Probe. C.J.L.M. Meijer has Honoraria of Speakers Bureau from GlaxoSmithKline and is a consultant/advisory board member for Qiagen. No competing interests were disclosed by the other authors.

## Authors’ contributions

GC and SF conceived and designed the study. GC, SF, IB, UT and DD were involved in the development of the methodology. DD (cytology), PJFS (HPV testing), CJLMM and MCGB (histological review), TG and MT (HPV testing) collected data. GC, IB, SF, and VT performed data analysis and interpretation. All authors read and approved the final manuscript. DD was responsible for administrative, technical, or material support. GC supervised the study.

## Pre-publication history

The pre-publication history for this paper can be accessed here:

http://www.biomedcentral.com/1471-2334/14/408/prepub
